# Correlates of species richness in the largest Neotropical amphibian radiation

**DOI:** 10.1111/j.1420-9101.2011.02243.x

**Published:** 2011-05

**Authors:** A Gonzalez-Voyer, J M Padial, S Castroviejo-Fisher, I De La Riva, C Vilà

**Affiliations:** * Department of Integrative Ecology, Estación Biológica de Doñana-CSICSevilla, Spain; † Department of Evolution, Genomics and Systematics, Evolutionary Biology Centre (EBC), Uppsala UniversityUppsala, Sweden; ‡ Departamento de Ciencias Biológicas, Instituto de Genética, Universidad de los AndesBogotá, Colombia; § Department of Biodiversity and Evolutionary Biology, Museo Nacional de Ciencias Naturales-CSICMadrid, Spain

**Keywords:** amphibian, diversification rates, montane gradients, phylogenetic comparative method, radiation, species richness, Terrarana

## Abstract

Although tropical environments are often considered biodiversity hotspots, it is precisely in such environments where least is known about the factors that drive species richness. Here, we use phylogenetic comparative analyses to study correlates of species richness for the largest Neotropical amphibian radiation: New World direct-developing frogs. Clade-age and species richness were nonsignficantly, negatively correlated, suggesting that clade age alone does not explain among-clade variation in species richness. A combination of ecological and morphological traits explained 65% of the variance in species richness. A more vascularized ventral skin, the ability to colonize high-altitude ranges, encompassing a large variety of vegetation types, correlated significantly with species richness, whereas larger body size was marginally correlated with species richness. Hence, whereas high-altitude ranges play a role in shaping clade diversity in the Neotropics, intrinsic factors, such as skin structures and possibly body size, might ultimately determine which clades are more speciose than others.

## Background

Evolutionary biologists have long been aware that species differ in their probabilities of diversifying or becoming extinct (Darwin, 1859) and that these differences are responsible for both the variation in the spatial pattern of species diversity (MacArthur & Wilson, 1967) and the disparate number of species across branches of the tree of life (Eldredge & Gould, 1972). However, evolutionary biologists still struggle to identify the mechanisms that determine species diversity, as well as their relative importance. This is especially evident in the tropics (reviewed in Moritz *et al.*, 2000; Mittelbach *et al.*, 2007), the most speciose environment but where least is known about the factors that influence among-clade differences in diversity. Identifying the mechanisms that have shaped species richness within highly diverse tropical environments will undoubtedly increase our understanding of worldwide patterns of species diversity (Wiens & Donoghue, 2004; Wollenberg *et al.*, 2008; Vences *et al.*, 2009). Moreover, present levels of extinction risk for tropical species (e.g. Stuart *et al.*, 2004) give a sense of urgency to studies aiming to expand current understanding of factors influencing speciation in these areas.

Differences among clades in the probability of diversifying are the result of a combination of contingent historical circumstances and intrinsic properties (Moore & Donoghue, 2007). On the one hand, it has been suggested that clade diversity largely depends on extrinsic historical factors influencing species ranges, such as episodes of climatic and habitat changes or orogeny (Ricklefs, 2003), as well as contingencies of the origin of the clade, such as latitudinal position (Ricklefs, 2006; Wiens, 2007; Svenning *et al.*, 2008). As an example, Smith *et al.* (2007) suggested that mid-altitude distribution ranges and early colonization of such areas enhance diversification of tree frogs, whereas Wiens *et al.* (2007) found that early colonization of mid-elevation habitats explain species richness patterns in salamanders. Kozak & Wiens (2007) found latitudinal differences in the altitudinal and climatic overlap of sister species, suggesting that climatic divergence along elevational gradients may increase opportunities for speciation and promote diversification in salamanders. On the other hand, intrinsic characteristics of the species may constitute potential key innovations (reviewed by Heard & Hauser, 1995). Body size (Purvis *et al.*, 2003), dispersal capabilities (Phillimore *et al.*, 2006; Moore & Donoghue, 2007), feeding generalization (Phillimore *et al.*, 2006) or a combination of life-history traits (Isaac *et al.*, 2005) have been found to be associated with species richness. Hence, extrinsic factors may provide the opportunity for diversification, whereas intrinsic species characteristics may determine whether such opportunities lead to moderate or explosive diversifications, extinction, or evolutionary stasis.

Rate of diversification has become a very important and widely used metric in macroevolutionary and macroecological studies (see Rabosky, 2009a,b;). However, recently the interpretation of estimates of rate of diversification, under certain circumstances, has been questioned (Rabosky, 2009a,b;). The critique centres on what has been suggested to be an underlying assumption of the distinct methods used to estimate rates of diversification, which is that species diversity increases unbounded through time (see Rabosky, 2009a). It has been suggested that when the underlying assumption of rates of diversification estimators is violated, rates might not be accurately estimated affecting the interpretation of the results (Rabosky, 2009a,b;). Under such circumstances, the focus of the study might need to be shifted away from factors influencing rates of diversification to factors influencing among-clade differences in species richness, if clade-age is not related to species richness (Rabosky, 2009a,b;). On the other hand, a possible influence of time on species richness might need to be accounted for if there is a relationship between clade age and species richness.

Here, we use phylogenetic comparative analyses to study correlates of species richness for the largest Neotropical amphibian radiation: New World direct-developing frogs. Identifying correlates of species diversity is an important first step towards understanding patterns of diversification in the tropics. Modern comparative methods are particularly useful for studying the factors associated with differences in species richness because they can incorporate information based on intrinsic species characteristics as well as properties of the environment in which they are found, all the while correcting for statistical nonindependence of data points because of phylogenetic relatedness (Felsenstein, 1985; Freckleton *et al.*, 2002).

**Fig. 1:**
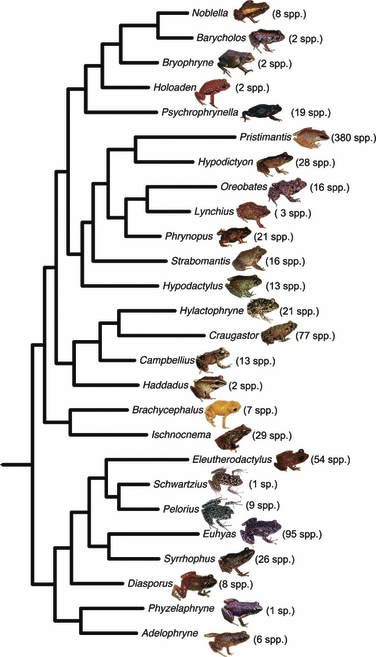
Phylogenetic relationships and species diversity for the 26 clades used in this study (modified from Hedges *et al.*, 2008; see Methods for more details about the tree). (Note: a colour version of this figure is available from Wiley Online Library.)

Terrarana, or New World direct-developing frogs, is an excellent example of extreme tropical species diversity. The clade Terrarana contains circa 900 species, which represent nearly 1/3 of all New World Tropics anuran species and nearly 1/6 of described anuran species worldwide (Hedges *et al.*, 2008). The pattern of species richness among clades within Terrarana is striking. The most recent phylogenetic hypothesis includes 26 clades ranked at the generic and subgeneric level, whose diversity varies from 1 to circa 380 species (Fig. 1; Hedges *et al.*, 2008). For example, the South American genus *Pristimantis*, with around 380 morphologically disparate species, constitutes one of the largest terrestrial vertebrate radiations reported to date (Heinicke *et al.*, 2007; Hedges *et al.*, 2008). Moreover, the members of this clade are important components of many Neotropical wet forests both in diversity and in abundance (Lynch & Duellman, 1997; Hedges *et al.*, 2008). In the West Indies, terraranans compose ca. 84% of the amphibian diversity (Hedges, 1999). Terraranans also present a very wide distributional range, spanning from the southern USA to northern Argentina, along a broad variety of habitats, from the cold paramos of the Andes at 4500 m.a.s.l. to Caribbean coastal forests (Hedges *et al.*, 2008). Unlike most amphibian species, which depend on water for reproduction (Vences & Köhler, 2008), terraranans undergo direct development without an aquatic larval phase (Duellman & Trueb, 1994), which allows species in this clade to live and reproduce in almost any environment given some moisture. Previously, the evolutionary history of this clade was poorly known. However, recent taxonomic and phylogenetic efforts have greatly improved our knowledge of the relationships among major clades within Terrarana (Crawford & Smith, 2005; Frost *et al.*, 2006; Hedges *et al.*, 2008; Heinicke *et al.*, 2009). Such recent developments in combination with existing data on phenotypic characters, distribution and habitat use provide an unprecedented opportunity to study the factors influencing clade diversity within this species-rich tropical clade.

The prevailing models to explain differences in clade diversity in Terrarana allocate a predominant role to extrinsic factors, including the Andean orogeny, the geological history of Middle America and Quaternary refugia (Lynch, 1986; Lynch & Duellman, 1997; Crawford & Smith, 2005; Heinicke *et al.*, 2007). However, such models alone cannot explain differences in species richness between clades of similar age, distributed in similar areas. Our aim here is to identify intrinsic and extrinsic factors associated with species richness among Terrarana clades. We use a mixed dataset combining intrinsic species characteristics (such as body size, body shape, ventral and dorsal skin characteristics, and morphological adaptations to arboreal or terrestrial habitats), as well as extrinsic variables (such as microhabitat, range size, altitude and latitude). We applied phylogenetic comparative methods to analyse rate of cladogenesis and to identify correlates of species richness of Terrarana using the most recent and most complete available phylogenetic and taxonomic information (Hedges *et al.*, 2008).

## Methods

### Phylogeny

We used the most complete (in terms of character and taxon sampling) published molecular phylogeny of Terrarana (Hedges *et al.*, 2008) for our analyses. Other studies deal with particular clades within Terrarana (Crawford & Smith, 2005; Padial *et al.*, 2008, 2009) or focus on more general phylogenetic relationships (Frost *et al.*, 2006). The phylogeny of Hedges *et al.* (2008) covers roughly 40% of ca. 900 species of Terrarana, and 26 of 32 generic and subgeneric taxa (the six missing supraspecific taxa together account for only 16 species – for which, there are no pertinent molecular data available). For the present study, we selected the best-supported tree from Hedges *et al.* (2008): a maximum likelihood phylogeny (ML tree) of 80 species based on 3709 base pairs (bp) of mitochondrial and nuclear DNA sequences, containing 26 nonoverlapping clades corresponding to the main genera and subgenera of Terrarana (Fig. 1). We used these 26 clades as our comparative units because they are the ones for which most phenotypic and natural history information was available, hence allowing us to maximize the number of characters included in our study. Species richness, the variable of interest, was assigned to each clade based on the taxonomy of Hedges *et al.* (2008) (Fig. 1, see also Table S2).

### Divergence times

We used the 80 species, 3709 bp matrix from Hedges *et al.* (2008) to estimate divergence times for the 26 taxa of interest in BEAST (Drummond & Rambaut, 2007) under a GTR + I + Γ substitution model. We applied the same four callibration points as those used by Heinicke *et al.* (2007) in their study: one in the outgroup (the split between *Agalychnis* callidryas and *Litoria caerulea*) and three in the ingroup. We ran the markov chain for 30 million iterations sampling every 1000 iterations with a burnin of 10%. We used a constant-rate Yule (speciation process) prior on the topology, a log-normal, uncorrelated relaxed clock prior for the substitution rate and uniform priors for the four callibration points. Convergence of the markov chain was verified in tracer v.1.5 (Rambaut & Drummond, 2007), and the effective sample size of all parameters was well above 200. To maintain uniformity with the published phylogeny of Hedges *et al.* (2008), we used this topology for annotation of node ages (see Table S2 for the list of estimated divergence times).

### Variables

We compiled a set of morphological and environmental variables from Hedges *et al.* (2008) for all clades (see Table S1). Generic and subgeneric clades of Terrarana usually show distinct morphological characters or combinations of them (e.g. related to skin texture and foot structures), and characters usually used by taxonomists may turn out to be adaptive (Hunter, 1998). We expected that some of the characters considered in our study might be adaptive characters that potentially increase species richness, either by favoring invasion of new adaptive zones, by enhancing ecological opportunity, by increasing fitness, or by promoting reproductive or ecological specialization (Heard & Hauser, 1995). The independent explanatory variables included were relative length of fingers and relative length of toes, presence of disc structures and plantar tubercles, ventral and dorsal skin characteristics, body shape, body size (maximum, minimum and size disparity, see Appendix S1 for details), as intrinsic variables, and microhabitat, maximum altitude, vegetation type diversity, latitude of distribution range and distributional range area, as extrinsic variables. However, we are aware that it is sometimes difficult to separate intrinsic and extrinsic variables. For example, the ecological gradient along which a clade is distributed can be considered an extrinsic factor promoting higher chances for allopatric speciation or as an intrinsic factor, relating to breadth of physiological tolerance. For a detailed description of variables, rationale of variable choice and coding strategy, see Supporting Information.

### Comparative analyses of the correlates of diversity

As mentioned in the introduction, it has been suggested that analyses of the correlates of diversification rate assume that species number within clades increases unbounded through time (Rabosky, 2009a,b;). If there is no relationship between species richness and rate of diversification, the interpretation of rates of diversification has been called into question and it has been suggested that the focus should turn to comparisons of clade richness (Rabosky, 2009a,b;). As previous studies of correlates of species richness among clades and regions estimated rates of diversification, we were interested in comparing the results of both metrics, also the results could serve as a guideline for researchers interested in analysing patterns of species richness among clades.

First, we analysed the relationship between the number of species within each clade (log-transformed) and the age of the clade (based on the stem age of the group, as crown ages could not be accurately estimated for some clades because of incomplete species coverage). If species richness within clades increases unbounded through time, then we could expect a positive relationship between clade age and species richness. We then proceded to calculate two variables, total clade diversification and rate of diversification, to compare the results between these two metrics and how these might be influenced if the underlying assumption of rate of diversification is violated. We calculated total clade diversification (Ω), as the log of species richness (Rabosky, 2009b). The net rate of diversification for a clade given its age was estimated first assuming negligeable extinction (Isaac *et al.*, 2003; Phillimore *et al.*, 2006) and second, based on the method-of-moments estimator of stem groups (Magallón & Sanderson, 2001), which explicitly incorporates non-neglieable extinction in the estimate of diversification rate. To test the robustness of results based on the method-of-moments estimator to uncertainty in the estimate of extinction, we used a range of estimates of relative diversity (species richness/extinction: ε = 0.00, 0.45, 0.90). Because the results did not vary if the analyses were undertaken with different estimates of epsilon, we only present results based on ε = 0.45 (see Table S3). Note that a recent simulation study showed that diversification rate estimates are not particularly sensitive to phylogenetic errors either in estimates of clade age or in topology (Wertheim & Sanderson, 2011). Finally, we tested whether the two estimates of rates of diversification were correlated with species richness, because if species richness is correlated with diversification rate estimates, then such rates should be relevant for explaining species richness patterns among clades (Kozak & Wiens, 2010).

We used phylogenetic generalized least squares (PGLS) multiple regression analyses to identify correlates of species richness and diversification rate (Martins & Hansen, 1997; Phillimore *et al.*, 2006). PGLS models account for shared evolutionary history of clades, and thus nonindependence of data points, by incorporating an estimate of the covariance of residuals resulting from shared ancestry in the error term (Martins & Hansen, 1997). The models also include the λ parameter whose maximum likelihood estimate corresponds to the transformation of the variance-covariance matrix of the linear model that best fits a Brownian motion model of evolution (Freckleton *et al.*, 2002).

Bivariate preliminary PGLS analyses showed that the following pairs of variables were correlated: relative length of fingers and relative length of toes, the logarithm of maximum altitude and vegetation type diversity, and body shape and microhabitat. To avoid multicolinearity, we combined each pair of correlated variables into a single component using phylogenetic principal components analysis (PPCA; Revell, 2009). Hence, relative length of fingers and relative length of toes were combined into a single variable, digit length. Vegetation type diversity and maximum altitude were combined into the variable vegetation – altitude. Body shape and microhabitat were combined into the variable microhabitat – shape. Note that maximum altitude and vegetation type diversity were significantly negatively correlated with the new variable vegetation – altitude, whereas all other variables presented positive correlations with their respective principal component. We also used PPCA to combine ventral and dorsal skin characteristics into a single component, skin texture. The first component was strongly negatively correlated with ventral skin and positively correlated, although less strongly, with dorsal skin*.* We thus included a total of nine explanatory variables in the multiple regression models (Table 1).

**Table 1:** Partial regression coefficients and standard errors (β ± SE), values of the statistic (*t*-value) and their associated significance value (*P*), for the complete multiple regression model including species richness (Ω) as the dependent variable (see text for details)

Trait	β ± SE	*T*-value	*P*
Intercept	−2.692 ± 2.397	−1.12	0.28
Disc structures	−0.498 ± 0.599	−0.83	0.42
Plantar tubercles	−0.501 ± 0.804	−0.624	0.54
Digit length	−0.090 ± 0.227	−0.40	0.70
Skin texture	**−0.891 ± 0.279**	**−2.91**	**0.01**
Maximum body size	1.937 ± 1.146	1.69	0.11
Microhabitat – shape	0.894 ± 0.660	1.36	0.20
Range size	0.601 ± 0.276	1.36	0.20
Vegetation – altitude	**−0.741 ± 0.316**	**−2.34**	**0.03**
Latitude	0.017 ± 0.026	0.66	0.52

Variables presenting a significant partial regression coefficient are highlighted in bold.

We used a backward stepwise elimination procedure to determine the minimal adequate model. Nonsignificant variables were removed in a stepwise fashion, choosing always the one with the least significant partial correlation coefficient. At every step, we compared the reduced model with the previous model by means of the small-sample version of the Akaike information criterion (AIC_c_), to determine whether model simplification resulted in a significant reduction in the variance explained (Burnham & Anderson, 2002). For all models, diagnostic plots were examined to check for normal distribution of errors and heteroscedasticity. All analyses were performed in R using PGLS code (Freckleton *et al.*, 2002) in the package CAIC.

## Results

### Clade-age, species richness and rates of diversification

**Fig. 2:**
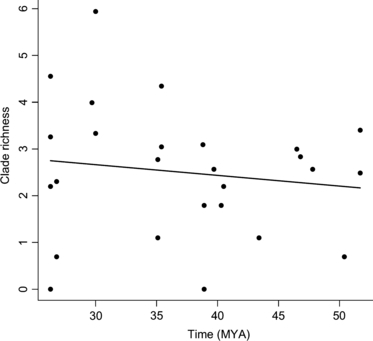
Correlation between log-transformed species richness and clade age for the 26 clades used in this study (phylogenetic generalized least squares: β = −0.03 ± 0.04, *t*_24_ = −0.88, *P* = 0.39).

We found a nonsignificant negative correlation between log-transformed species richness and the age of the clade (PGLS: β = −0.03 ± 0.04, *t*_24_ = −0.88, *P* = 0.39; Fig. 2), even when incorporating phylogenetic information. The negative correlation between species richness and clade-age suggests that contrary to what would be predicted under unbounded diversification within clades, older clades are not more species-rich than younger clades. The two estimates of rate of diversification (diversification rate and method-of-moments estimator) were significantly correlated with species richness, even when correcting for phylogenetic effects (β = 28.03 ± 1.73, *t*_24_ = 16.21, *P* < 0.0001 and β = 32.04 ± 2.01, *t*_24_ = 15.92, *P* < 0.0001, respectively). These results suggest that diversification rate is a relevant metric, even if the suggested assumption is violated. High diversification rates in young clades may decouple diversification rates from patterns of species richness, in any case the highly significant correlation between species richness and rate of diversification suggests that, even if diversification rates change over time, variation in rates of species accumulation nonetheless capture differences in species richness among the clades studied (Kozak & Wiens, 2010).

Furthermore, the results are generally the same whether we used clade species richness, net rate of speciation or the method-of-moments estimator as our dependent variable. The only differences with the model including species richness were the value of *R*^2^ and minor quantitative differences in parameter estimates (see Table 3 and Table S3). For brevity we only present the results of the analyses using total clade diversification (Ω) as our dependent variable and the results of the minimum model of the analyses using net rate of diversification as the dependent variable (but see Table S3).

**Table 3:** Partial regression coefficients and standard errors, values of the statistic (*t*-value) and their associated signficance value (*P*), for the minimum adequate model using net rate of speciation as the dependent variable. The model explained 64% of the variance in net rate of diversification (*R*^2^ = 0.64)

Trait	β ± SE	*T*-value	*P*
Intercept	−0.10 ± 0.06	−1.69	0.11
Skin texture	**−0.01 ± 0.007**	**−2.12**	**0.05**
Maximum body size	0.06 ± 0.03	1.99	0.06
Range size	0.01 ± 0.007	1.84	0.08
Vegetation – altitude	**−0.03 ± 0.01**	**−3.88**	**0.001**

Variables presenting a significant partial regression coefficient are highlighted in bold.

### Correlates of species richness

The complete model including all independent variables (Table 1) explained 71% of the variance in total clade diversification and was highly significant (*F*_16,26_ = 3.69, *P* = 0.01). The maximum likelihood value of the λ parameter was 0.0007, however it was neither significantly different from 0 nor from 1 (χ^2^ = −0.0004, *P* = 0.99, and χ^2^ = 1.29, *P* = 0.26). Three variables presented significant partial regression coefficients: skin, range size and vegetation – altitude (note that as vegetation and altitude both correlated negatively with the first component, the results of the analyses actually suggest that both variables correlate positively with diversification rate; see Table 1). The minimum adequate model explained 65% of the variance and was highly significant (*F*_21,26_ = 9.64, *P* = 0.0001). Four variables were retained in the minimum model: skin, maximum body size, range size and vegetation – altitude; of these, maximum body size and range size presented only marginally significant partial regression coefficients (Table 2). The maximum likelihood λ estimate was the same as above. The results were generally the same when we used rate of diversification as the dependent variable (see Table 3).

**Table 2:** Partial regression coefficients and standard errors (β ± SE), values of the statistic (*t*-value) and their associated significance value (*P*), for the minimum adequate model including species richness (Ω) as the dependent variable (see text for details)

Trait	β ± SE	*T*-value	*P*
Intercept	−2.315 ± 1.705	−1.36	0.19
Skin texture	**−0.575 ± 0.203**	**−2.83**	**0.01**
Maximum body size	1.660 ± 0.849	1.956	0.06
Range size	0.347 ± 0.196	1.77	0.09
Vegetation – altitude	**−0.759 ± 0.200**	**−3.80**	**0.001**

Variables presenting a significant partial regression coefficient are highlighted in bold.

To gain better understanding of the influence of skin characters, we analysed ventral and dorsal skin characters separately as categorical traits in the phylogenetic minimum model. These analyses indicated that it is the aerolate ventral skin character that is associated with higher total clade diversification as it was this category, which differed significantly from the others with respect to total clade diversification (*t*_21_ = 2.56; *P* = 0.019). Dorsal skin, on its own, was not significantly correlated with total clade diversification. Furthermore, when we replaced maximum body size with either minimum body size or body size disparity in the minimum model, neither of the two later variables showed a significant correlation with clade richness (β = −0.590 ± 1.076, *t*_21_ = −0.55, *P* = 0.59 and β = −0.406 ± 0.384, *t*_21_ = −1.06, *P* = 0.30, respectively).

## Discussion

### Clade-age, species richness and diversification rates

The nonsignificant negative correlation between log-transformed species richness and clade age suggests that age differences among terraranan clades are not sufficient to explain differences in species richness. Different factors have been put forward to explain the absence of a relationship between clade age and species richness.

First, reduced variation in clade age among compared groups might lead to other factors having a stronger influence on species richness. However, clade ages in Terrarana are relatively different, varying from 26.3 to 51.7 MYA (see Table S2). Note also that simulations suggest that heterogeneity among clades in rate of diversification is not sufficient to eliminate a positive association between clade age and species richness (Rabosky, 2009a).

Second, a correlated speciation-extinction dynamic, whereby clades with the highest net diversification rate tend to have higher extinction rates, might explain the observed temporal pattern in species richness. Such a positive speciation-extinction correlation, dubbed clade ‘volatility’ (Gilinsky, 1994), has received support from paleontological studies (Stanley, 1979; Gilinsky, 1994; Liow *et al.*, 2008). The clade ‘volatility’ hypothesis predicts that only younger clades would show high extant species richness because older clades would be pruned because of extinction. The three most speciose clades in Terrarana are relatively young (26.3–30 MYA) as would be expected under clade ‘volatility’. However, a recent simulation study suggests that clade volatility is unlikely to explain the null correlation between age and diversity observed in higher taxa (Rabosky, 2009a). More importantly, our results show that both ecological factors and intrinsic species characteristics are significantly associated with species richness, and such variables explain a high proportion of the variance in clade richness than clade age. Thus, although we cannot discard the possibility that extinction in older clades contributed to shape clade richness in Terrarana (in addition, the fossil record in Terrarana is practically inexistent), our results indicate that additional factors have contributed to increase diversity in certain clades.

Third, departures from an age-diversity correlation could be explained by ecological factors constraining species richness (McPeek, 2008; Rabosky, 2009a) or species characteristics influencing rates of diversification (e.g. Purvis *et al.*, 2003; Isaac *et al.*, 2005; Phillimore *et al.*, 2006). The ecological limits hypothesis (Rabosky, 2009b) predicts that species richness accumulates during the early stages of diversification (or the first stages of a radiation) when there is still an ample availability of unexploited niches. Once the niche space has been filled, speciation declines and speciation-extinction dynamics leads clade diversity to stasis. The negative correlation between species richness and clade age in Terrarana could thus be explained by ecological factors having constrained diversification in older clades. Analyses of diversification patterns in molecular phylogenies have found patterns consistent with ecological constraints on species richness (e.g. Nee *et al.*, 1992; Sepkoski, 1998; Harmon *et al.*, 2003; Weir, 2006; Price, 2008; Phillimore & Price, 2008; Rabosky & Lovette, 2008; Rabosky, 2009a). On the other hand, differences between older and younger clades in their rates of diversification, influenced by species characteristics, could also explain the negative age – diversity correlation.

Interestingly, even though the nonsignificant negative clade-age species richness correlation violates the assumption of unbounded rates of diversification within clades (Rabosky, 2009a,b;), our results are the same whether we use species richness or rate of diversification as the dependent variable (see Table 3 and Table S3). It is not surprising that the results do not differ between analyses undertaken with total clade diversification (Ω) or rate of diversification as these metrics were significantly and strongly correlated. Based on these results, we propose a rough guidline for the choice of an adequate metric for analyses of correlates of diversity. First, test whether clade-age and species richness are correlated. If the correlation is positive, we suggest the most appropriate metric is rate of diversification as the effect of time-for-speciation (e.g. Stephens & Wiens, 2003) needs to be controlled. If the correlation between clade-age and species richness is negative, the correlation between species richness and rates of diversification should be analysed (e.g. Kozak & Wiens, 2010). The two metrics will probably be correlated in a majority of cases, as correcting for clade age will result in higher rate of diversification in younger than older clades. If clade richness and rate of diversification are indeed correlated, choice of the most appropriate metric will depend on the question being addressed and knowledge of the natural history of the group under study. Finally, if there is no relationship between clade-age and species richness, and rates of diversification are uncorrelated (or negatively correlated) with species richness, estimates of rates of diversification could be biased (Rabosky, 2009a,b;); hence, we suggest the appropriate metric under such circumstances is total clade diversification.

### Correlates of diversification in Terrarana

The phylogenetic multiple regression model explained a high proportion of the variance in species richness (Freckleton, 2009). The minimum adequate model including only four independent variables explained 65% of the variance in species richness (Table 2). This model indicates that Terraranan clades with the ability to colonize high-altitude ranges and encompassing different vegetation types are more speciose than clades restricted in altitude and occupying few vegetation types. A recent study has found that higher rates of species diversification are associated with higher rates of climatic-niche evolution, especially in the tropics (Kozak & Wiens, 2010). Additionally, an increase in ventral skin vascularization was positively correlated with species richness. Maximum body size and range size are apparently also important explanatory variables in the multiple regression model, even though their partial regression coefficients were not significant. However, other traits, such as latitude or morphological characters associated with arboreal dwelling, were not significantly correlated with species richness.

### Latitude and range size

The latitudinal position of clades has been considered an important variable to explain differences in speciation and/or extinction rates between tropical and temperate regions (e.g. Ricklefs, 2006; Wiens, 2007; Svenning *et al.*, 2008). However, we found no significant correlation between latitude and species richness. Even though Terrarana mostly has a Neotropical distribution, ranges do span from southern USA to northern Argentina, as mentioned above (Hedges *et al.*, 2008). Previous support for the latitudinal effect on diversification hypothesis is mixed. For example, Wiens (2007) and Moore & Donoghue (2007) found that diversification rates increases towards low latitudes in amphibians and Dispsacales (plants). On the other hand, Wiens *et al.* (2006, 2009) found that both extinction and diversification rates were similar in tropical and temperate clades, suggesting that neither an acceleration of speciation in the tropics nor greater temperate extinction rates explain high tropical diversity in hylid and ranid frogs. Recently, Weir & Schluter (2007) found that for birds and mammals, both speciation and extinction correlate positively with an increase in latitude and that this fast turnover at higher latitudes mediates the latitudinal species gradient (but see Tobias *et al.*, 2008). An important artefact of using the midpoint of the latitudinal position of a clade as a proxy for the ancestral geographical location is that current distribution, especially the midpoint, does not necessarily reflect past distribution (Losos & Glor, 2003). Indeed, some old and poorly diverse clades, such as *Adelophryne*, *Holoaden* and *Noblella*, show fragmented distributions across large areas, which may suggest that currently known species are remnants of once more diverse and broadly distributed clades.

Range size has long been considered a factor intimately related to diversification (MacArthur & Wilson, 1967) and/or allopatric speciation (Rosenzweig, 1978). An association between range size and species richness could have been expected because clades of Terrarana with very different diversity occupy areas that greatly differ in size both on the continent (*Pristimantis* in South America and *Craugastor* in Central America) and in archipelagos (*Euyhas* and *Eleutherodactylus* in the Caribbean). In addition, the most diverse clades also occur along environmental and altitudinal gradients, which, coupled with large distribution ranges, would increase the chance for vicariance. In spite of such theoretical and practical predictions, we did not find a significant correlation betwen the variable range size and species richness, although there was a positive tendency (see Fig. S1). This probably reflects the fact that the vast majority of clades and species of Terrarana are distributed along mountain ranges in which a high turnover of habitats and changes in selective pressures over short distances occur because of the complex orography and differences in climate (Lynch & Duellman, 1997; Kozak & Wiens, 2010).

### Morphological traits and diversification rates

Species of Terrarana usually show distinct and conspicuous morphological characters or combinations of them (Hedges *et al.*, 2008; Duellman & Lehr, 2009) – e.g. related to skin texture and finger and toe structures, size – which have been traditionally used by taxonomists to delimit species. Such character differences could, at least theoretically, have an adaptive origin (Hunter, 1998). Indeed, Hedges (1999) suggested that the morphological diversity of *Eleutherodactylus* from Jamaica, and probably also in the Greater Antilles, was the result of an adaptive radiation. We therefore expected that our results would highlight potential adaptive characters associated with high species richness.

Only one morphological trait was significantly correlated with species richness, skin characteristics, although body size was marginally significantly correlated. Ventral skin presented a strong negative loading on the first principal component of the skin characteristics PPCA, whereas dorsal skin presented a low positive loading, suggesting that the first component explains more variance in ventral skin. Furthermore, when analysed separately, only ventral skin was significantly correlated with species richness, and the analyses suggest that it is a more aerolate ventral skin that is positively associated with higher species richness. This implies that the association between species richness and skin characteristics is mostly driven by the presence of an aerolate (probably more vascularized) ventral skin. Amphibian skin is involved in respiration, osmoregulation and thermoregulation (Duellman & Trueb, 1994). Hence, variation in skin structures may be important for adaptation to different environments. In addition, our results show an association between species richness and higher vascularization in ventral skin and altitude (and vegetation types). We speculate that more vascularized bellies might have proven advantageous during colonization of high altitude ranges (potentially reflecting an adaptation to lower atmospheric oxygen levels) and hence increasing species richness. A possible avenue for future research could be to analyse the relationship between skin structures and osmotic regulation and respiration in high altitude habitats.

Body size may be involved in niche partitioning and has long been considered an important variable to explain differences in species diversity (Lomolino, 1985; Purvis *et al.*, 2003; Pfennig & Pfennig, 2005; Moen & Wiens, 2009; but see Adams *et al.*, 2009). For example, it has been suggested that small-bodied species may diversify more than larger ones because they can produce a more fine-grained division of niche space (Lomolino, 1985; Purvis *et al.*, 2003). This is a long-standing hypothesis that has received some support in amphibians. For example, Lynch & Duellman (1997) reported evidence for a correlation between small body size, arboreal habits and species richness in *Pristimantis* (the clade with highest number of Terraranan species). Duellman (2005) found that prey size was strongly correlated with body size in a tropical community and suggested that competitive release by resource partitioning could explain the high local diversity of frogs in the Amazon, up to 139 species in 6.5 km^2^ (Bass *et al.*, 2010). Also, in the Caribbean tree-frogs genus *Osteopilus*, prey size was strongly associated with body size, which suggests that body-size divergence has facilitated resource partitioning and diversification of these frogs (Moen & Wiens, 2009). Also, larger species are more resistant to evaporative water loss and better able to maintain body heat (Shoemaker, 1992). Hence, species with different body sizes might partition habitats based on humidity and ambient temperature, with smaller species being usually restricted to more mesic habitats, potentially resulting in more species-rich clades presenting larger disparity in body size. Nonetheless, our models do not support the hypothesis that clades with smaller species or with higher disparity in body size also present higher species richness.

There was no *a priori* hypothesis associating higher species richness with large body size, which complicates the explanation for the pattern at hand. We suggest that the observed pattern may be the result of a higher capacity of larger frogs to disperse and colonize new habitats because larger body size could be adaptive for higher evaporative water loss or low temperatures in high-altitude habitats. Indeed, Hedges (1999) reported a positive correlation between body size and altitude in arboreal species of Jamaica and the Greater Antilles. However, when comparing among Terrara clades, maximal body size of a clade was not correlated with the maximum altitude at which species in the clade are found (β = −0.00001 ± −0.00003, *t*_24_ = −0.42, *P* = 0.67). The ability of species to expand their ranges seems to play an important role in diversification (Moore & Donoghue, 2007; Moyle *et al.*, 2009). For example, van Bocxlaer *et al.* (2010) report that the highest diversification rates of toads (family Bufonidae) were coupled with colonization of new areas, and this happened shortly after toads evolved traits facilitating dispersal, such as increased body size. Thus, clades with larger species might have been able to disperse and inhabit areas with different environments and selective pressures, which might have led to higher species richness. This suggestion is supported by the fact that preliminary analyses showed that clades with a higher altitudinal distribution were also present in a diversity of vegetation types. On the other hand, when comparing among clades, maximal body size is not correlated with diversity of vegetation types (β = 0.026 ± 0.044, *t*_24_ = 0.60, *P* = 0.56).

### Mountain gradients and diversification

Our finding that clade diversity is favoured by both altitude and vegetation gradients has important implications for understanding patterns of diversity both within Terrarana – and hence most of the Neotropical realm – and for tropical montane areas in general. Most studies aiming to explain the origin of montane diversity can be grouped under three basic models.

Under the first model, diversification occurs in sympatry or parapatry. Mountain areas produce more diversity because they create more ecological gradients and divergent selection on species-specific characteristics drives lineage diversification (Endler, 1977; reviewed by Benton, 2009; and Schluter, 2009). This model rests on the assumption that morphologically different sister species in sympatry or in adjacent altitudinal belts originated through divergence with gene flow. However, it is usually difficult to discern whether phenotypic divergence originated in allopatry, or if disruptive selection acting on ecomorphological traits produced speciation (Rundell & Price, 2009). Although this model cannot be discussed at the taxonomic scale of our study, it is important to note that Lynch & Duellman (1997) found that many of the altitudinally segregated species of *Pristimantis* were putative sister taxa (although without a phylogenetic framework). And also Elmer *et al.* (2007) and Padial & De la Riva (2009) for *Pristimantis*, and Hua & Wiens (2010), for *Eleutherodactylus*, reported sister species occupying contiguous vegetation types segregated either altitudinal or latitudinally. Those cases constitute good candidates to assess the possible contribution of the divergence with gene flow model to the diversification of mountain faunas.

The second model relies on extrinsic factors and proposes that geographical isolation associated with mountain building and/or recurrent climatic changes account for most of the diversity (Graves, 1985; Patton & Smith, 1992; Colinvaux, 1993; Fjeldså, 1994; Rahbek, 1997; Hughes & Eastwood, 2006; Weir, 2006; Cadena *et al.*, 2007; Ribas *et al.*, 2007). However, if only extrinsic factors (vicariance/dispersal events) were at play in the diversification of Terrarana, we would expect (i) groups sharing distribution ranges and time of origin to have approximately the same number of species and (ii) clades showing a broad altitudinal distribution across ecological gradients to be highly diverse. This is not the case, at least for some groups with large distribution along broad altitudinal gradients that show low species richness (e.g. *Hypodictyon*). This supports the suggestion that species-specific characteristics determine how they respond to the geographical drivers of diversification (Crawford *et al.*, 2007; Moyle *et al.*, 2009). Indeed, those particular differences are what give sense to a third, mixed model, the one that has predominated discussions about the diversification of Terrarana (Lynch & Duellman, 1997).

Under the third model, geographical isolation drives speciation, but divergent selection is what keeps species evolving along different trajectories after secondary contact (Lynch, 1986; Lynch & Duellman, 1997). Under this model, groups with more capacity to speciate will minimize the effect of extinction, and rapidly diverging species will avoid species fusion after secondary contact. This seems to be the case of highly diverse groups of Terrarana, favoured by intrinsic characteristic, as important morphological traits uncover in this study or other overlooked traits favouring the colonization of high altitude habitats and vegetational belts. Both identifying historical factors promoting speciation and additional traits that have allowed some groups to diversify more than others is an aspect that surely deserves future research.

## Conclusions

Younger clades of Terrarana have more species than older ones, which contradicts the longstanding time for speciation hypothesis for this group. The pattern may be explained by ecological constraints to diversification in older clades coupled with ongoing high speciation in younger clades (potentially favoured by higher ecological opportunity in these young clades), without neglecting a possible role for extinction. Our results suggest that whereas montane gradients may play a critical role in increasing clade diversity (either by creating geographical isolation, different selective pressures or, most probably, a combination of both) in the Neotropics, phenotypic traits, such as presence of skin structures and body size might be ultimately determining which clades have more species than others. Our study highlights the importance of considering both intrinsic and extrinsic factors in the search for causes of differences in species richness among clades. Future studies could consider analysing whether body size and skin characteristics represent adaptations associated with life in high altitude ranges. Such studies could provide further support to the hypothesis that certain speciose Terraranan clades constitute undiscovered adaptive radiations.

## Supporting information

**Figure S1** Partial regression graph of the relationship between total clade richness (Ω) and log-transformed range size within the minimum adequate model.

**Table S1** Extrinsic and intrinsic variables for different clades used for comparative phylogenetic analyses.

**Table S2** Recognized number of species per clade, clade age, diversification rate [Log(species richness)/clade age], and product of moment estimator (with ε = 0.45) for different clades.

**Table S3** Minimum adequate model using method-of-moments estimator as the dependent variable (*R*^2^ = 0.63).

**Appendix S1** Variable description, rationale for variable choice and variable coding, and datamatrixs for comparative phylogenetic analyses.

As a service to our authors and readers, this journal provides supporting information supplied by the authors. Such materials are peer-reviewed and may be reorganized for online delivery, but are not copy-edited or typeset. Technical support issues arising from supporting information (other than missing files) should be addressed to the authors.
