# Supplementary material for: Correlates of species richness in the largest Neotropical amphibian radiation
Source: J Evol Biol. 2011 May;24(5):931–42. doi: 10.1111/j.1420-9101.2011.02243.x (PMC3116151; doi:10.1111/j.1420-9101.2011.02243.x)
Supplement: Supplementary file 1 [file jeb0024-0931-SD1.doc]

**Appendix S1.** Variable description, rationale for variable choice and variable coding, and datamatrixs for comparative phylogenetic analyses (Tables S1 and S2).

*Hand and foot structures*—Anuran amphibians specialized in arboreal habitats show several sets of adaptations that involve changes in relative proportions or in the degree of development of finger structures (Duellman and Trueb 1994). The main traits distinguishing Terraranans with arboreal habits from those with terrestrial habits are the modified finger and toe pads associated to climbing (Pough et al. 2002). However, the plantar structures called plantar tubercles might also be important for diversification due to their potential adaptive value in particular environments. Clades with eminently terrestrial species possess well-developed and irregular conical turbecles in the plantar surfaces of feet and hands (eg. *Oreobates*, see Padial et al. 2008), which may provide necessary adherence to the moist leaf litter floor to perform quick movements for prey capture or to escape from predators. On the other hand, arboreal species rarely present well-developed plantar tubercles, which could become cumbersome when trying to grab branches or to stand on leaves. Another set of traits that might enhance adaptation to arboreal or terrestrial habitats is the relative length of fingers and toes. Within Terrarana, arboreal species usually present a shorter thumb relative to the second finger, while the opposite seems to occur in clades with mostly terrestrial species. A pattern that has proven more difficult to explain is that of the relative length of toes V and III. The adaptive value of this character is unknown, but it could also be related to arboreal or terrestrial habits. We assigned a value of 1 to clades containing species with disc structures (variable Disc Structures), and a value 2 to those lacking disc structures. Clades with prominent Plantar Tubercules received a value of 1, those with moderately developed tubercles received a value of 1.5, and those with low or no tubercles received a value of 2. Relative lengths of finger I and finger II were classified in the following manner (values in parentheses): loss of fingers (0); I>II (1), I=II (2), I<II (3), variable (4). Relative length of toes III and V were classified as follows: loss of toes (0); III>V (1); III=V (2), III<V (3).

*Skin texture*—The skin of amphibians is permeable to water and plays an important role in respiration, osmoregulation and termoregulation (Duellman and Trueb, 1994). Skin texture has been largely used for defining supraspecific taxa (e. g., Lynch and Duellman 1997). Terraranans inhabiting a riparian environment generally present a completely smooth belly, while more arboreal or terrestrial species have aerolate bellies providing more vascularized surface (Duellman and Trueb 1994). Skin texture varies from completely smooth to coarsely aerolate, with most Terraranans falling in one or the other of the two extremes. Developing structures for protection or enhancing water absorption and gas exchange such as granules, folds or vascularized surfaces may result in increased physiological performance. We divided dorsal and ventral skin texture into two categorical variables: smooth (1), polymorphic (2), and tuberculate (3), which capture the main general patterns found in Terrarana.

*Body size*— It has been suggested that small-bodied species may diversify more than large species because they can produce a more fine-grained division of niche space (Lomolino 1985; Purvis et al. 2003). Indeed, shifts in body size between species can result from character displacement associated to resource competition (Pfenning and Pfenning 2005). On the other hand, frogs with larger body size generally have higher resistance to evaporative water loss (Schoemaker 1992) and species with different body sizes might partition habitats based on humidity, with smaller species being usually restricted to more mesic habitats, potentially resulting in more species-rich clades presenting larger disparity in body size. In this study we considered body shape as well as three distinct measures of body size as potentially influencing diversification. For body shape we distinguished between robust frogs (value = 1) and slender ones (2). We included the maximum body size of the members of a clade, the minimum size, and the disparity in body size (calculated as maximum size – minimum body size). Maximum and minimum body sizes were Log 10 transformed. The different body size measures were entered in the model in a sequential fashion to avoid problems of multicolinearity. However, it must be noted that the measures provide different information, as our analyses showed that disparity in body size was not significantly correlated with maximum body size or with minimum body size (R^2^ = 0.04, F _24, 26_ = 2.06, p = 0.16; and R^2^ = 0.01, F _24, 26_ = 0.18, p = 0.68, respectively), even while controlling for phylogeny.

*Microhabitat*—Microhabitat was codified based on the use of terrestrial or arboreal environments. This information is highly relevant in combination with the aforementioned morphological characters, which have been suggested to reflect adaptations to arboreal life or terrestriality (e.g. disc structures, plantar tubercles). Clades were coded as terrestrial (value= 1), arboreal (2), or both (1.5). The latter value was assigned when species numbers within a clade were roughly the same for arboreal and terrestial microhabitat. In cases where only a few species within the clade differed from the general pattern, the clade was codified based on the general pattern.

*Geographic range size*—Large range size has long been considered a correlate of species richness and diversification rates (MacArthur and Wilson 1967) because the environment may be more hererogeneus in larger areas, thus facilitating niche partitioning (Losos and Schluter 2000) or because large areas may increase the chances for allopatric speciation (Rosenzweig 1978). Geographic range sizes were calculated under an equal area projection in ArcMap for ArcGIS 9.2 (ESRI 2006) using distribution maps of Hedges et al. (2008), which are based on individual species maps from the Global Amphibian Assessment database (S. B. Hedges, pers. com.; see range maps on IUCN, 2010).

*Vegetation type diversity*—In a gradient model of diversification, strong environmental differences associated with altitude (e.g. vegetation types) may result in adaptive divergence and speciation (Endler 1977). Terrarana species are mainly forest inhabitants, with some clades adapted to the upper limit of cloud forests (“Ceja de montaña”) and to paramos, while others range from the lowlands to ca. 4000 m.a.s.l. Vegetation types along altitudinal gradients in South and Central America can be grouped into four main categories: lowland forests, montane forests, cloud forests and paramos (Richards et al. 1996). Clades were assigned values of 1–4 depending of the number of the vegetation types they occupied. Since the altitude where each one of these vegetation types is found depends on latitude, slope orientation, and other factors, this discrete coding scheme does not account for variance in altitudinal distributions, with which we deal below, but captures what would be considered the range of vegetation types in which species from the clade are found (e.g. Brumfield and Edwards 2007).

*Altitude*— Several studies show that altitude is related to clade diversity because montane regions are usually subjected to vicariance events that provide opportunities for allopatric speciation (Graves 1985; Colinvaux 1993; Fjeldså 1994; Lynch and Duellman 1997; Fjeldså and Rahbek 2006; Weir 2006; Cadena et al. 2007) and because environmental changes associated with higher altitudes may lead to adaptive divergence with gene-flow along ecological gradients (García-París et al. 2000; Graham et al. 2004; Roberts et al. 2006; Elias et al. 2009). We calculated maximum altitude for a clade from the ranges of altitudinal distribution in Hedges et al. (2008). This variable was Log 10 transformed.

*Latitude*— Global patterns of amphibian diversity suggest that the latitudinal variation in species richness (most species are found in the tropics) might be the result of differences in speciation and/or extinction rates between tropical and temperate regions (Wiens 2007)—although the high tropical diversity of some clades cannot be explained by this hypothesis (e.g. Wiens et al. 2006). To explore if differences in clade diversity within Terrarana are associated to the latitudinal position of clades, we calculated the midpoint between the northernmost and southernmost localities for each clade following Wiens (2007).

| Clades | Discs | Tubercles | Fingers | Toes | Ventral skin | Dorsal skin | Body | Med size | Max size | Microhabitat | Vegetation | Max  altitude | Range  Size | Latitude |
| --- | --- | --- | --- | --- | --- | --- | --- | --- | --- | --- | --- | --- | --- | --- |
| Adelophryne | 1.0 | 1.5 | 3.0 | 1.0 | 1.0 | 1.0 | 2.0 | 40.5 | 64 | 1.0 | 1.0 | 1000 | 195907 | -5 |
| *Barycholops* | 1.0 | 1.0 | 1.0 | 1.0 | 1.0 | 1.0 | 1.0 | 31 | 31 | 1.0 | 1.0 | 600 | 52891 | -4 |
| *Brachycephalus* | 1.0 | 2.0 | 0.0 | 0.0 | 3.0 | 1.0 | 1.0 | 18 | 18 | 1.0 | 1.0 | 1500 | 382051 | -18 |
| *Bryophryne* | 1.0 | 1.5 | 3.0 | 2.0 | 3.0 | 3.0 | 1.0 | 29 | 29.3 | 1.0 | 2.0 | 4120 | 862 | -13 |
| *Campbellius* | 2.0 | 1.5 | 1.0 | 1.0 | 1.0 | 3.0 | 1.0 | 43.5 | 65 | 1.0 | 3.0 | 2000 | 37155 | 14.5 |
| *Craugastor* | 2.0 | 1.5 | 1.0 | 1.0 | 2.0 | 2.0 | 1.0 | 64 | 110 | 1.5 | 3.0 | 2700 | 1102205 | 15 |
| *Diasporus* | 2.0 | 1.5 | 3.0 | 3.0 | 3.0 | 1.0 | 2.0 | 18.5 | 26 | 2.0 | 3.0 | 2500 | 202597 | 7.25 |
| *Eleutherodactylus* | 2.0 | 1.0 | 3.0 | 3.0 | 3.0 | 1.0 | 2.0 | 48.5 | 80 | 2.0 | 3.0 | 2000 | 184218 | 19 |
| *Euhyas* | 2.0 | 1.0 | 3.0 | 1.0 | 1.0 | 2.0 | 1.0 | 37.5 | 64 | 1.0 | 3.0 | 2000 | 180321 | 20.5 |
| *Haddadus* | 2.0 | 1.5 | 1.0 | 2.0 | 2.0 | 2.0 | 1.0 | 40.5 | 64 | 1.0 | 1.0 | 1200 | 514963 | -20 |
| *Holoaden* | 1.0 | 1.5 | 1.0 | 1.0 | 3.0 | 3.0 | 1.0 | 48 | 48 | 1.0 | 1.0 | 2000 | 26696 | -24 |
| *Hylactophryne* | 2.0 | 1.5 | 3.0 | 3.0 | 1.0 | 3.0 | 1.0 | 56 | 95 | 2.0 | 3.0 | 2000 | 867753 | 22.5 |
| *Hypodactylus* | 1.0 | 1.5 | 1.0 | 2.0 | 1.0 | 2.0 | 1.0 | 32.5 | 49 | 1.0 | 3.0 | 3700 | 17049 | -2 |
| *Hypodictyon* | 2.0 | 1.0 | 4.0 | 3.0 | 2.0 | 2.0 | 2.0 | 44 | 72 | 2.0 | 3.0 | 2000 | 1249265 | 7 |
| *Ischnocnema* | 2.0 | 1.0 | 2.0 | 1.0 | 2.0 | 2.0 | 1.0 | 35 | 54 | 1.0 | 1.0 | 1200 | 676421 | -18 |
| *Lynchius* | 1.0 | 1.5 | 1.0 | 3.0 | 1.0 | 1.0 | 1.0 | 43 | 43 | 1.0 | 2.0 | 3100 | 1043535 | -5 |
| *Noblella* | 1.0 | 1.5 | 3.0 | 3.0 | 1.0 | 3.0 | 1.0 | 22 | 22 | 1.0 | 3.0 | 3500 | 982008 | -9 |
| *Orebates* | 2.0 | 1.0 | 1.0 | 1.0 | 1.0 | 2.0 | 1.0 | 41.5 | 63 | 1.0 | 3.0 | 2800 | 1479943 | -11 |
| *Pelorius* | 2.0 | 1.0 | 1.0 | 3.0 | 1.0 | 1.0 | 1.0 | 68 | 88 | 1.0 | 2.0 | 1700 | 74305 | 19 |
| *Phrynopus* | 1.0 | 1.0 | 3.0 | 3.0 | 2.0 | 2.0 | 1.0 | 34.3 | 54 | 1.0 | 2.0 | 4400 | 23379 | -9.5 |
| *Phyzelaphryne* | 1.0 | 1.0 | 2.0 | 1.0 | 1.0 | 3.0 | 2.0 | 20 | 20 | 1.0 | 1.0 | 100 | 952060 | -4 |
| *Pristimantis* | 2.0 | 1.5 | 3.0 | 3.0 | 3.0 | 2.0 | 2.0 | 43 | 73 | 2.0 | 3.0 | 3300 | 7496009 | -3 |
| *Psychrophrynella* | 1.0 | 1.5 | 3.0 | 1.0 | 3.0 | 2.0 | 1.0 | 23.5 | 33 | 1.0 | 2.0 | 4200 | 26354 | -15.5 |
| *Schwartzius* | 2.0 | 1.0 | 3.0 | 1.0 | 1.0 | 1.0 | 2.0 | 57 | 57 | 1.0 | 1.0 | 800 | 3260 | 19 |
| *Strabomantis* | 2.0 | 1.5 | 1.0 | 1.0 | 1.0 | 3.0 | 1.0 | 68 | 106 | 1.0 | 3.0 | 2400 | 1097347 | 0 |
| *Syrrhophus* | 2.0 | 1.0 | 2.0 | 1.0 | 2.0 | 2.0 | 2.0 | 51 | 83 | 1.0 | 3.0 | 2400 | 846487 | 22 |

**Table S1**. Extrinsic and intrinsic variables for different clades used for comparative phylogenetic analyses. See material and methods for a detailed explanation of variables. (Variable abbreviations: Discs = Disc structures, Tubercles = Plantar tubercles, Fingers = Relative length of fingers, Toes = Relative length of toes, Body = Body shape, Med size = Median body size, Max size = Maximum body size, Vegetation = Vegetation type diversity, Max Alt = Maximum altitude).

| TAXA | Species  diversity | Clade  Age (Myr) | Diversification  rate | Product  moment estimator |
| --- | --- | --- | --- | --- |
| *Adelophryne* | 6 | 38.9 | 1.792 | 0.046 |
| *Barycholos* | 2 | 26.8 | 0.693 | 0.026 |
| *Brachycephalus* | 12 | 51.7 | 2.485 | 0.048 |
| *Bryophryne* | 6 | 40.3 | 1.792 | 0.044 |
| *Campbellius* | 13 | 39.7 | 2.565 | 0.065 |
| *Craugastor* | 77 | 35.4 | 4.344 | 0.123 |
| *Diasporus* | 9 | 40.5 | 2.197 | 0.054 |
| *Eleutherodactylus* | 54 | 29.7 | 3.989 | 0.134 |
| *Euhyas* | 95 | 26.3 | 4.554 | 0.173 |
| *Haddadus* | 2 | 50.4 | 0.693 | 0.014 |
| *Holoaden* | 3 | 43.4 | 1.099 | 0.025 |
| *Hylactophryne* | 21 | 35.4 | 3.045 | 0.086 |
| *Hypodactylus* | 13 | 47.8 | 2.565 | 0.054 |
| *Hypodictyon* | 28 | 30 | 3.332 | 0.111 |
| *Ischnocnema* | 30 | 51.7 | 3.401 | 0.066 |
| *Lynchius* | 3 | 35.1 | 1.099 | 0.031 |
| *Noblella* | 10 | 26.8 | 2.303 | 0.086 |
| *Oreobates* | 16 | 35.1 | 2.773 | 0.079 |
| *Pelorius* | 9 | 26.3 | 2.197 | 0.084 |
| *Phrynopus* | 22 | 38.8 | 3.091 | 0.080 |
| *Phyzelaphryne* | 1 | 38.9 | 0.000 | 0.000 |
| *Pristimantis* | 380 | 30 | 5.940 | 0.198 |
| *Psychrophrynella* | 20 | 46.5 | 2.996 | 0.064 |
| *Schwartzius* | 1 | 26.3 | 0.000 | 0.000 |
| *Strabomantis* | 17 | 46.8 | 2.833 | 0.061 |
| *Syrrhophus* | 26 | 26.3 | 3.258 | 0.124 |

**Table S2**. Recognized number of species per clade, clade age, diversification rate (Log(species richness)/clade age), and product of moment estimator (with ε = 0.45) for different clades.

| **Trait** | **β ± se** | **T-value** | **p** |
| --- | --- | --- | --- |
| Intercept | -0.10 ± 0.05 | -1.83 | 0.08 |
| **Skin texture** | **-0.014 ± 0.006** | **-2.12** | **0.05** |
| Maximum Body Size | 0.053 ± 0.03 | 2.00 | 0.06 |
| Range Size | 0.011 ± 0.006 | 1.87 | 0.08 |
| **Vegetation – Altitude** | **-0.02 ± 0.006** | **-3.64** | **0.002** |

**Table S3.** Minimum adequate model using method-of-moments estimator as the dependent variable (R^2^ = 0.63)


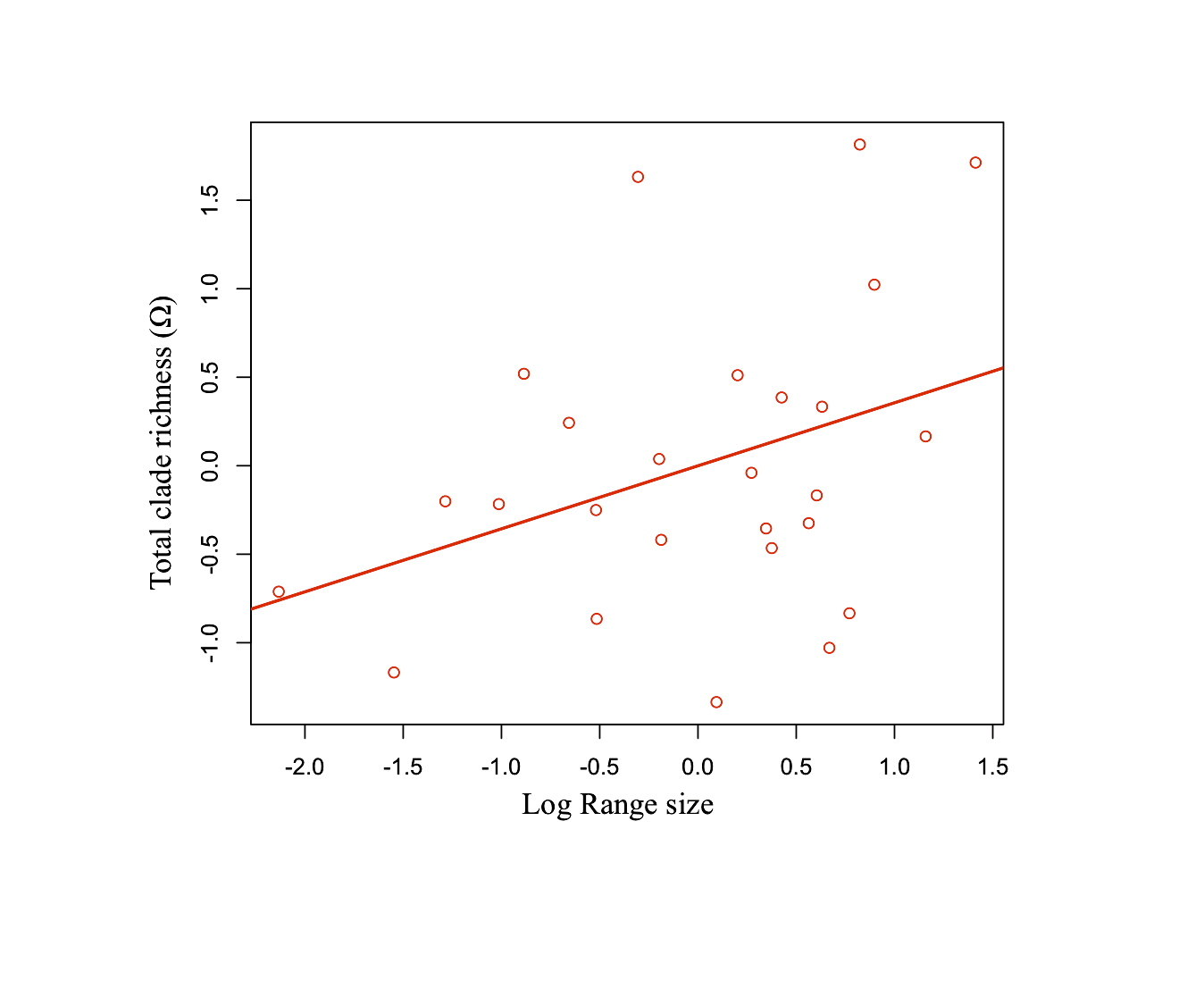


**Figure S1.** Partial regression graph of the relationship between total clade richness (Ω) and log-transformed range size within the minimum adequate model (see Table 2 and Methods for details). Note that the figure is plotted without controlling for phylogenetic relationships among clades.
